# Editorial: Crosstalk between cell death, oxidative stress, and immune regulation

**DOI:** 10.3389/fimmu.2024.1503252

**Published:** 2024-10-29

**Authors:** Chung-Nga Ko, Chao Yang

**Affiliations:** ^1^ Department of Chemistry, Hong Kong Baptist University, Hong Kong, Hong Kong SAR, China; ^2^ National Engineering Research Center for Marine Aquaculture, Institute of Innovation & Application, Zhejiang Ocean University, Zhoushan, China

**Keywords:** cell death, oxidative stress, immune regulation, immune-mediated diseases, apoptosis

Cell death, a basic physiological process of all organisms, involves a series of core players capable of destroying the homeostasis of cellular environment. With more thorough research in recent years, different types of cell death such as apoptosis, autophagy, necroptosis and ferroptosis have been clarified (1–3). This Research Topic compiles a range of contributions exploring the intricate relationships among cell death, oxidative stress, and immune regulation, as well as their pathobiology and therapeutic implications in immune-mediated diseases.

The review from Liu et al. summarized the pre-clinical and clinical studies of the pathogenesis of transfusion-related acute lung injury (TRALI). In the presence of stimuli, neutrophil extracellular traps (NETs) are formed by activated neutrophils and are established as effector molecules, contributing to the release of ROS that destroys pulmonary vascular endothelial cells. The authors discussed the mechanism through which NETs induce TRALI, and highlighted the possible therapeutic targets based on the modulation of NETosis/NETs, for example, through activation of the glycolytic pathway, targeting inflammasome, chemokines/cytokines and neutrophil receptors. Another review from Zhang et al. described the role of cGAS-STING pathway in viral infection. Apart from its most common function in regulating IFN-α and inflammation, cGAS-STING also has major impacts on a series of cellular responses, such as endoplasmic reticulum stress, autophagy and oxidative stress. However, overactivation and inactivation of the cGAS-STING pathway are both detrimental to the clearance of pathogens. Further studies on how to modulate the activity of cGAS-STING and promote elimination of virus by host cells are still required.

This Research Topic also focuses on the therapeutic strategies targeting the “cell death-oxidative stress-immune regulation” signaling axis. For example, the research article from Dos Santos et al. reported a repurposed drug deucravacitinib, which is a tyrosine kinase 2 (TYK2) inhibitor, for the prevention and treatment of type 1 diabetes. The result shows that deucravacitinib could prevent the effects of IFN-α in a dose-dependent manner while not affecting the function and survival of β-cells. In cells pre-treated with proinflammatory cytokines, deucravacitinib could partially reduce inflammation and apoptosis. This pre-clinical data suggests that TYK2 inhibition may be an effective strategy for treating type 1 diabetes. Another review article from Zhang et al. reported the research progress of mesenchymal stem cells-derived extracellular vesicles (MSC-EVs) and exosomes (MSC-Exos), which carry bioactive molecules e.g. regulatory proteins and miRNA, in the treatment of oxidative stress-related diseases. The regulatory activities of MSC-EVs and MSC-Exos, including apoptosis, necrosis and oxidative stress, on many systemic diseases have been widely validated by cellular and animal models. However, the same bioactive molecules in MSC-EVs and MSC-Exos seem to have different effects in different studies. It is therefore necessary to formulate a protocol to better control the isolation steps of MSC-EVs and MSC-Exos, as well as to select study models closer to human pathology for better clinical usage. The review from Mackiewicz et al. discussed the role of nuclear factor of activated T-cells (NFAT), which is a family of main transcription factors responsible for regulating the expression of genes important for inflammatory and immune responses, in Alzheimer’s diseases. The inflammatory mediators produced by NFAT-dependent pathway is controlled by Ca^2+^-dependent protein phosphatase calcineurin (CaN) and aberrant NFAT-CaN signaling may play a deleterious role in the pathologies of Alzheimer’s diseases, including neuronal apoptosis. Although targeted inhibition of CaN/NFAT may offer a promising strategy in the treatment of Alzheimer’s diseases, the severe adverse effects of many CaN inhibitors and scarce research on NFAT inhibitors have markedly limited their translational potential. Another review from Maiese discussed three pathways of programmed cell death, including SIRT1, AMPK and WISP1, and suggested that these pathways are potentially important in maintaining nervous system function and metabolic homeostasis, which warrant more thoughtful research.

The study of gene regulatory networks is useful to understand transcriptional dynamics in biological systems. Computational recognition of regulator genes has been successfully applied to study the relationship between programmed cell death/oxidative stress and different diseases. The research article from Xu et al. explored the patho-physiobiological mechanisms underlying atopic dermatitis (AD). The authors identified 278 differentially expressed genes (DEGs) and seven ferroptosis signature genes in four AD-related cohorts from the GEO database (samples from patients with AD and healthy controls). Four ferroptosis genes (*EGR1, MAP3K1, FABP4, ALOXE3*) were selected to construct a FerrSig predictive model and was shown to be able to accurately identify patients at higher risks of AD. Another research conducted by Li et al. integrated single-cell RNA sequencing and bulk transcriptomic datasets to elucidate the mechanisms underlying renal ischemia-reperfusion injury (RIRI). The authors identified five necroptosis-related DEGs from the pre- and post-reperfusion renal biopsies using gene expression data, constructed a predictive model for delayed graft function (DGF) and divided patients into different risk groups. The model revealed reliable performance in identifying patients with higher risks of developing DGF. The result was further validated by mouse models that exhibited up-regulated necroptosis-related DEGs after ischemia-reperfusion. The same research group (Zhang et al.) conducted another study identifying three endoplasmic reticulum stress-related genes (*ATF3, JUN* and *PPP1R15A*) which were found to be associated with different kidney injury-related pathways, including apoptosis, pyroptosis, oxidative stress and inflammatory response. Intriguingly, compared with the sham group, the expression of the three genes were significantly higher after RIRI, and were decreased after treatment with a potential drug for RIRI. Furthermore, a research article from Yang et al. established three transcriptional co-expression networks (clusters C1, C2 and C3) with distinct antioxidative potential in glioblastoma cancer cells. C2, which was identified as a cluster with a moderate level of ROS, was found to exhibit a strong correlation with the highly aggressive mesenchymal subtype of glioblastoma. Among the transcriptional factors in C2, *FOSL1* demonstrates a prognostic value in both overall survival and overall-free interval.

In summary, therapeutic strategies targeting the pathways of cell death offer exciting prospects for maintaining homeostasis of cellular environment that can be compromised in immune-mediated diseases. Integrating predictive models with other clinical indicators may also provide a comprehensive assessment to identify patients with higher risks of disease development/recurrence and can potentially offer a promising prognostic application to alleviate disease burden. We hope that this Research Topic will enrich our understanding of the crosstalk between cell death, oxidative stress, and immune regulation, and will open up new avenues for the diagnosis and treatment of immune-mediated diseases.
